# Post-Training Dephosphorylation of eEF-2 Promotes Protein Synthesis for Memory Consolidation

**DOI:** 10.1371/journal.pone.0007424

**Published:** 2009-10-13

**Authors:** Heh-In Im, Akira Nakajima, Bo Gong, Xiaoli Xiong, Takayoshi Mamiya, Elliot S. Gershon, Min Zhuo, Ya-Ping Tang

**Affiliations:** 1 Department of Cell Biology and Anatomy, Louisiana State University Health Sciences Center, New Orleans, Louisiana, United States of America; 2 Neuroscience Center of Excellence, Louisiana State University Health Sciences Center, New Orleans, Louisiana, United States of America; 3 Department of Physiology, Faculty of Medicine, University of Toronto, Toronto, Ontario, Canada; 4 Departments of Psychiatry and Human Genetics, the University of Chicago, Chicago, Illinois, United States of America; Max-Planck-Institut fuer Neurobiologie, Germany

## Abstract

Memory consolidation, which converts acquired information into long-term storage, is new protein synthesis-dependent. As protein synthesis is a dynamic process that is under the control of multiple translational mechanisms, however, it is still elusive how these mechanisms are recruited in response to learning for memory consolidation. Here we found that eukaryotic elongation factor-2 (eEF-2) was dramatically dephosphorylated within 0.5–2 hr in the hippocampus and amygdala of mice following training in a fear-conditioning test, whereas genome-wide microarrays did not reveal any significant change in the expression level of the mRNAs for translational machineries or their related molecules. Moreover, blockade of NMDA receptors with MK-801 immediately following the training significantly impeded both the post-training eEF-2 dephosphorylation and memory retention. Notably, with an elegant sophisticated transgenic strategy, we demonstrated that hippocampus-specific overexpression of eEF-2 kinase, a kinase that specifically phosphorylates and hence inactivates eEF-2, significantly inhibited protein synthesis in the hippocampus, and this effects was more robust during an “ongoing” protein synthesis process. As a result, late phase long-term potentiation (L-LTP) in the hippocampus and long-term hippocampus-dependent memory in the mice were significantly impaired, whereas short-term memory and long-term hippocampus-independent memory remained intact. These results reveal a novel translational underpinning for protein synthesis pertinent to memory consolidation in the mammalian brain.

## Introduction

The process of learning and memory may be divided into several sequential steps, including acquisition, consolidation, storage, and retrieval [1], [2]. As the initial step, acquisition requires the brain to be at a higher arousal level in order to acquire new information as much as possible. However, only a very small portion of the acquired information may be further processed in the brain for long-term storage, a process that is called memory consolidation [3]. Consolidated memory traces are then transferred to certain brain regions such as the cortex for long-term storage. As consolidated information is retrievable over hours, days, months, and even up to the whole lifetime, this type of memory, called long-term memory, plays an essential role in human intelligent life. In contrast, information that does not undergo a consolidating process can only last for seconds or minutes. This type of memory is called short-term memory [4], [5]. Without any doubt, to explore the molecular and neuronal mechanisms underlying memory consolidation is not only fundamental for our understanding of how acquired information is encoded in the brain but also insightful for disclosing how long-term memory formation could be impaired even though the acquisition is normal. This is of particular interest, as this kind of mnemonic dysfunction is often observed in many pathological conditions and clinical entities such as abnormal aging, mental retardation, and an early stage of neurodegenerative disease such as Alzheimer's disease [6]–[8].

A milestone over the past century for the studies of learning and memory is the demonstration that *de novo* new protein synthesis is required for memory consolidation [9], [10], although recent evidence indicates that post-translational modifications of certain existing proteins may also be important for early consolidation [11]. This milestone has been established based essentially on the studies with the use of pharmacological/neurosurgical approaches. For example, post-training infusion of a protein synthesis inhibitor such as anisomycin into the hippocampus, amygdala, and motor cortex of the animals significantly impairs hippocampal, emotional, and motor memory, respectively [12]–[15]. An important advantage of the use of those approaches is the feasibility for both the temporal-specificity (within a special time window during the process of learning and memory) and spatial-specificity (targeting on a particular brain region), both of which allow a real-time/on-site coupling analysis of learning behavior in free-moving animals. However, as protein synthesis itself is a series of complicated biochemical reactions, it is still unclear how a neuronal process (learning) bridges to these biochemical reactions in the brain.

In mammalian cells, protein synthesis is mediated by interactions between a target mRNA and translational machineries including ribosomal proteins, eukaryote initiation factors (eIFs), and eukaryote elongation factors (eEFs). Upon mRNAs available, eIFs such as eIF4E recognize and bind to a target mRNA, and then eEFs such as eEF-2 mediate the polypeptide elongation [16], [17]. Importantly, the activities of these translational machineries are regulated by many other molecules such as eEF-2 kinase (eEF-2K), eIF-binding proteins, etc. [16], [17], all of which are called “translational machinery-related molecules” here. An important regulatory mechanism for most of these molecules is phosphorylation/dephosphorylation. Hyperphosphorylation of eIF4E-binding protein-1 by mammalian target of rapamycin (mTOR), for example, activates eIF4E, and consequently, promotes protein synthesis [18], [19]. Phosphorylation of eEF-2 by eEF-2K inactivates eEF-2 and therefore, significantly inhibits protein synthesis [20], although the effect of eEF-2K might depend on certain condition in an *in vitro* system [21]. Originally known as Ca^2+^-calmodulin-dependent protein kinase III [22], eEF-2K is present in all cells in the body [23]. It is therefore reasonably to speculate that an alteration in either the expression level or the phosphorylation state of these translational machineries or their related molecules including eEF-2K may significantly change the process of protein synthesis, which in turn may facilitate or impede a particular long-lasting biological process such as memory consolidation.

In this report, we provide compelling evidence at the molecular, pharmacological, genetic, and behavioral levels that post-training dephosphorylation of eEF-2 is a key translational mechanism for protein synthesis pertinent to memory consolidation in the brain.

## Results

### Post-training expression of mRNAs for translational machineries and their related molecules in brains of mice following training in a fear-conditioning test (FCT)

**Figure 1:**
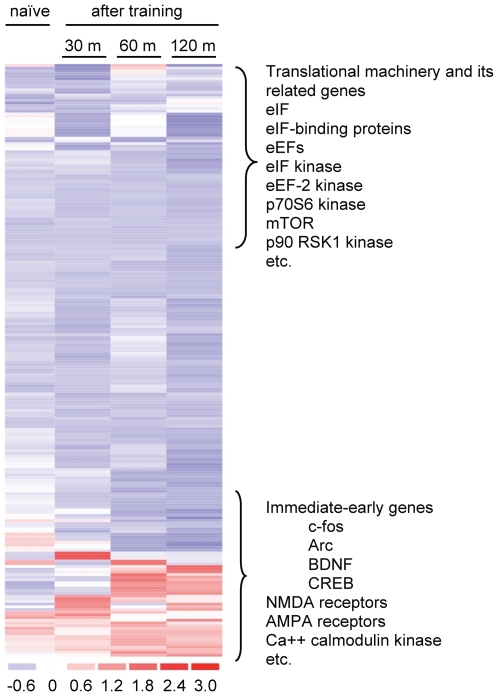
Gene expression profiles in the hippocampus of B6/CBA F1 mice following the training in FCT. A time-course of 30, 60, and 120 min (m) was examined. Over 100 probes that detected mRNAs that encode to transcriptional machineries or their related molecules and neuronal activity were used. No significant change in the expression level of translational machineries or their related molecules was found, whereas a number of neuronal activity-related genes were either up- or down-regulated, of which many immediate-early genes were up-regulated.

The FCT is the most commonly used behavioral paradigm for the studies of long-lasting fear memory in the rodent [24]. In order to determine whether training in the FCT altered the expression of translational machineries or/and their related molecules in the brain regions that are critically involved in consolidating fear memory, genome-wide cDNA microarrays were used to screen gene expression profiles in the hippocampus, amygdala, and cortex. A time-course of 30, 60, and 120 min after the training, together with a control group, was examined. As shown in Figure 1 and Table 1, some neuronal activity-related genes such as c-fos, BDNF, and Arc were up-regulated in the hippocampus of mice after the training, whereas the expression of mRNAs for ribosome, eIFs, eIF-binding proteins, eEFs, eIF kinase, eEF-2K, mTOR, p70S6 kinase, and p90 RSK1 kinase, etc. was not significantly changed, in comparison with those in control mice. Similar results were observed in the amygdala and cortex (data not shown), indicating that changes in the expression level of the mRNAs for the translational machineries and their related molecules are unlikely to be a mechanism that is pivotal for memory consolidation-associated protein synthesis.

**Table 1:** Expression profiles of translational molecules and their related molecules in the hippocampus of B6/CBA F1 mice after training in a fear-conditioning test.

Gene Name	Profiles
eukaryotic translation initiation factor 3 (Eif3)	0.8
eukaryotic translation initiation factor 4E binding protein 2 (Eif4ebp2)	1.2
eukaryotic translation initiation factor 2, subunit 3, structural gene Y-linked (Eif2s3y)	1.1
eukaryotic translation initiation factor 4E binding protein 1 (Eif4ebp1)	0.9
eukaryotic translation initiation factor 2, subunit 2 (beta, 38kDa)	1.0
Similar to eukaryotic translation initiation factor 3, subunit 4 (delta, 44kD)	1.0
eukaryotic translation initiation factor 2A (Eif2a)	1.5
eukaryotic translation initiation factor 2 alpha kinase 4 (Eif2ak4)	1.2
eukaryotic translation initiation factor 2, subunit 3, structural gene X-linked (Eif2s3x)	1.5
heme-regulated eIF2 alpha kinase (Hri)	1.1
eukaryotic translation initiation factor 2 alpha kinase 1	1.0
eukaryotic translation initiation factor 2 alpha kinase 2	1.8
eukaryotic translation initiation factor 4E	1.0
eukaryotic translation initiation factor 1A	1.1
eukaryotic translation initiation factor 3, subunit 5 (epsilon)	1.0
eukaryotic translation initiation factor 4A1	1.1
Highly similar to e2be rat translation initiation factor eIF-2B epsilon subunit	1.0
eukaryotic translation initiation factor 3, subunit 3 (gamma, 40kD)	0.4
eukaryotic translation initiation factor 4E binding protein 1	1.4
eukaryotic translation initiation factor 4A1	1.0
eukaryotic translation initiation factor 4E binding protein 2	0.9
eukaryotic translation initiation factor 5A	1.0
eukaryotic translation initiation factor 4A2	1.0
eukaryotic translation initiation factor 3, subunit 2 (beta, 36kD) (Eif3s2)	1.1
eukaryotic translation initiation factor 3	1.1
eukaryotic translation initiation factor 2, subunit 2 (beta, 38kDa) (Eif2s2)	1.1
eukaryotic translation initiation factor 2 alpha kinase 3 (Eif2ak3)	1.0
eukaryotic translation initiation factor 2B (Eif2b)	1.2
eukaryotic translation initiation factor 4E	1.1
eukaryotic translation initiation factor 4A2	1.0
eukaryotic translation initiation factor 5A	1.0
Mouse RNA-dependent EIF-2 alpha kinase	1.8
eukaryotic translation initiation factor 2A	1.2
eukaryotic translation initiation factor 2 alpha kinase 1	1.3
eukaryotic translation initiation factor 4E binding protein 2	1.1
eukaryotic translation initiation factor 3, subunit 8 (110 kDa)	0.9
eukaryotic translation initiation factor 2, subunit 2 (beta, 38kDa)	1.0
Highly similar to S72266 translation initiation factor eIF2B gamma chain	0.9
eukaryotic translation initiation factor 5A2	1.3
eukaryotic translation initiation factor 3, subunit 8 (110 kDa) (Eif3s8)	1.1
eukaryotic translation initiation factor 3, subunit 8 (110 kDa)	0.9
eukaryotic translation initiation factor 4, gamma 2 (Eif4g2)	1.1
eukaryotic translation initiation factor 3, subunit 3 (gamma, 40kD) (Eif3s3)	1.0
eukaryotic translation initiation factor 3, subunit 7 (zeta, 6667 kDa) (Eif3s7)	0.8
eukaryotic translation initiation factor 3, subunit 2 (beta, 36kD) (Eif3s2)	0.9
eukaryotic elongation factor, selenocysteine-tRNA-specific (Eefsec)	1.3
eukaryotic translation elongation factor 1 alpha 2 (Eef1a2)	0.9
eukaryotic translation elongation factor 1 alpha 1	0.9
eukaryotic translation elongation factor 2	1.0
eukaryotic translation elongation factor 1 beta 2	1.0
eukaryotic translation elongation factor 1 beta 2 (Eef1b2)	1.0
eukaryotic elongation factor-2 kinase	1.2
eukaryotic translation elongation factor 1 epsilon 1 (Eef1e1)	1.1
eukaryotic translation elongation factor 1 delta	1.0

### Dephosphorylation of eEF-2 (dephospho-eEF-2) in both the hippocampus and amygdala was temporarily associated with post-training

**Figure 2:**
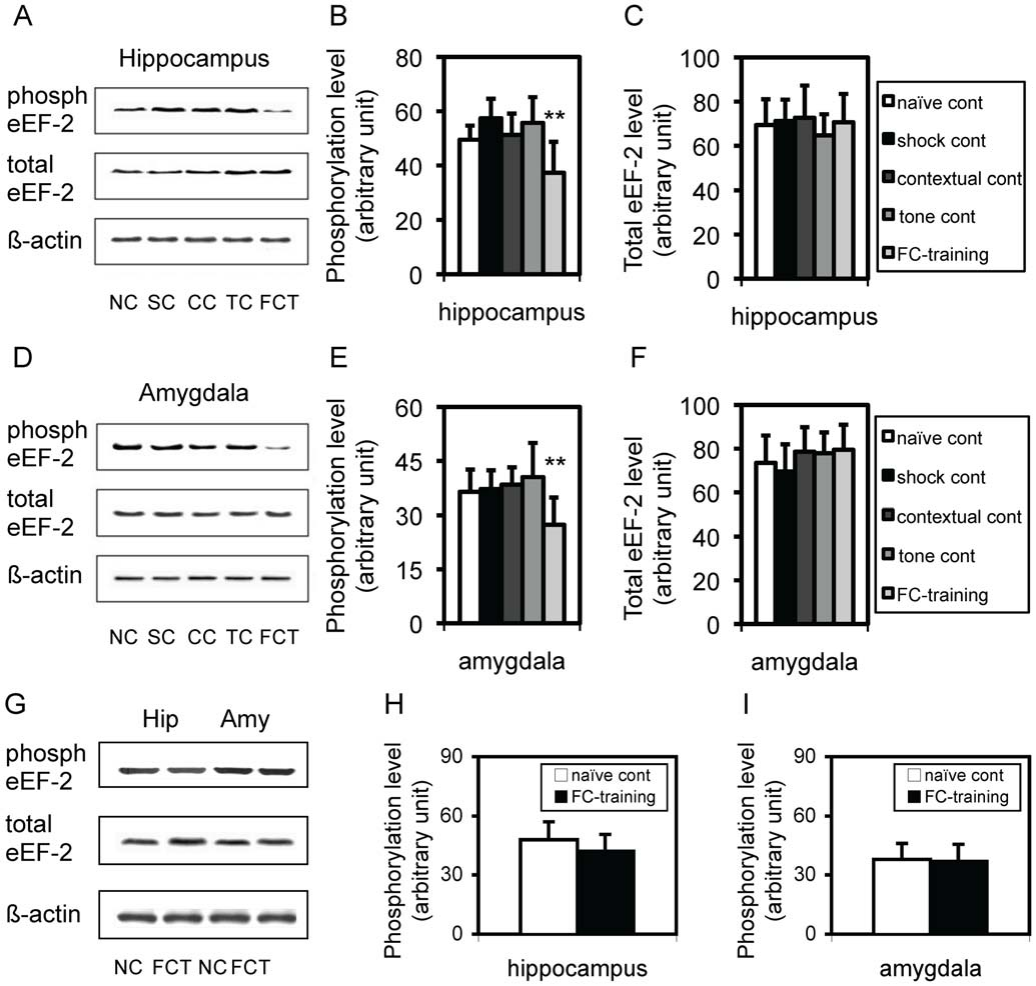
Dephospho-eEF-2 in both the hippocampus and amygdala is temporally associated with post-training. A. Representative Western blots showing the expression level of phospho-eEF-2 (phosph eEF-2; upper panel), total eEF-2 (middle panel), and β-actin (low panel) in hippocampi from mice that were sacrificed 30 min after the training. NC: naïve control; SC: shock control; CC: contextual control; TC: tone control; FCT: fear-conditioning training. B. Quantitative analysis of phospho-eEF-2 in hippocampi from mice that were sacrificed 30 min after the training (n = 5), in comparison to NC (n = 6), SC (n = 5), CC (n = 5), and TC (n = 5). C. Quantitative analysis of the total eEF-2 level in hippocampi of the same mice as described in B. D. Representative Western blots showing the expression level of phospho-eEF-2 (phosph eEF-2; upper panel), total eEF-2 (middle panel), and β-actin (low panel) in amygdalae from the same mice as described in B. E. Quantitative analysis of phospho-eEF-2 in amygdalae from the same mice as described in B. F. Quantitative analysis of the total eEF-2 level in amygdalae of the same mice as described in B. G. Representative Western blots showing the expression level of phospho-eEF-2 (phosph eEF-2; upper panel), total eEF-2 (middle panel), and β-actin (low panel) in both hippocampi (Hip) and amygdalae (Amy) from mice that were sacrificed 4 hr after the training. (H). Quantitative analysis of phospho-eEF-2 in hippocampi from NC (n = 6) and trained mice (n = 5) that were sacrificed 4 hr after the training in FCT. (H). Quantitative analysis of phospho-eEF-2 in amygdalae from the same mice as described in H. **, p<0.01, one-way ANOVA.

We next focused on whether the phosphorylation of eEF-2 (phospho-eEF-2) in brains of mice altered after the training in FCT. A time-course of 30 min, 2 hr, and 4 hr was examined. In order to exclude any non-specific effect, four control conditions, naïve control (NC), shock control (SC), contextual control (CC), and tone control (TC), were examined. At 30 min, the level of phospho-eEF-2 in the hippocampus (Figure 2A), amygdala (Figure 2D), but not the cortex (data not shown), was dramatically decreased, compared to that in the same brain regions of control mice. After normalization to the NC level, about 25–35%, 24–38%, and 3–7% of eEF-2 was dephosphorylated in the hippocampus (Figure 2B), amygdala (Figure 2E), and cortex (data not shown), respectively, and an ANOVA revealed a significant difference in dephospho-eEF-2 between trained and control mice in either the hippocampus [F(1,4) = 7.39; p<0.01] or amygdala [F(1,4) = 9.07; p<0.01], but not in the cortex. Post-hoc tests revealed significant differences (p<0.05 or 0.01) between trained group and every control group, but not between any two control groups. For the total eEF-2 level (phospho-eEF-2 and dephospho-eEF-2), neither an observable difference (Figure 2A and D) nor a statistical significance (Figure 2C and F) was noted in the hippocampus (Figure 2A and C), amygdala (Figure 2D and F), or cortex (data not shown) between trained and control mice. Moreover, at 2 hr after the training, a very similar pattern of dephopho-eEF-2 and the total eEF-2 level to those observed at 30 min after the training was found in both the hippocampus and amygdala, together with no any significant change in both of the dephopho-eEF-2 and total eEF-2 level in the cortex (data not shown). Four hours after the training, the dephospho-eEF-2 in the hippocampus (Figure 2G and H) and amygdala (Figure 2G and I) returned to the pre-training level, indicating that there was a time window for the dephosphorylation. Similarly, the phospho-eEF-2 in the cortex and the total eEF-2 level in all these three brain regions were not significantly different between trained and control mice. All these results indicated that the dephospho-eEF-2, but not the change in its expression level, in both the hippocampus and amygdala was temporally but significantly associated with post-training while this change was not noted in the cortex.

### MK-801 concurrently blocked memory consolidation and post-training dephospho-eEF-2

**Figure 3:**
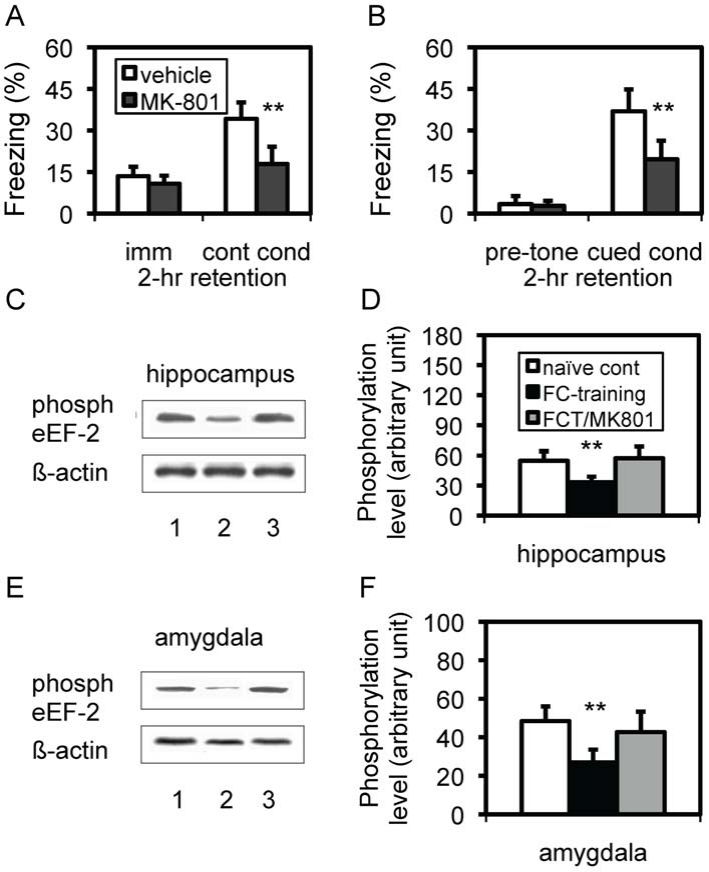
Effect of MK-801 on post-training dephospho-eEF-2 and memory retention. A. Contextual conditioning 2 hr after training/MK-801 treatment. **, p<0.01, Student's *t* test. Imm: immediate freezing; cont cond: contextual conditioning. B. Cued conditioning memory in the same mice. **, p<0.01, Student's *t* test. cued cond: cued conditioning. C. Expression level of phospho-eEF-2 (phosph) in hippocampi from mice 2 hr after training/MK-801 treatment. 1: naïve control; 2: mice with training/vehicle; 3: mice with training/MK-801. D. Quantitative analysis of the expression of phospho-eEF-2 in hippocampi from mice 2 hr after the training. **, p<0.01; Student's *t* test, compared between trained group-treated with vehicle and either naïve group or trained group-treated with MK-801. E. The expression level of phospho-eEF-2 (phosph) in amygdalae from mice 2 hr after the training. The same mice, as described above, were used. F. Quantitative analysis of the expression of phospho-eEF-2 in amygdalae of mice 2 hr after training. **, p<0.01; Student's *t* test, compared between trained group-treated with vehicle and either naïve group or trained group-treated with MK-801.

Post-training dephospho-eEF-2 provided a clue for us to explore whether this change served as a working mechanism for memory consolidation. We first addressed whether there was a functional link. Based on the essential role of the NMDA receptor in memory consolidation [25], [26], we examined whether antagonism of NMDA receptors could block both memory consolidation and dephospho-eEF-2. Mice were treated with MK-801 (0.2 mg/kg; i.p.) immediately following the training, and memory retention and phospho-eEF-2 were examined 2 hr thereafter. To exclude an acute effect of MK-801, another group of mice was examined for their memory retention 24 hr after the treatment. As expected, mice treated with MK-801 were significantly impaired in both contextual and cued conditionings at either 2 hr (Figure 3A and B) or 24 hr (data not shown), compared to those in mice treated with vehicle (p<0.01; Student's t test). Since the treatment (i.p.) lacked a brain region-specificity, the overall mnemonic function was affected. Based on our previous findings [25] and others [27], [28], together with the effect observed at 24 hr here, this impairment should attribute to a deficit in memory consolidation. Very interestingly, the same MK-801-treatment prevented the post-training dephospho-eEF-2 in the hippocampus (Figure 3C) and amygdala (Figure 3E) at 2 hr after the treatment. A statistical significance was observed in the hippocampus (Figure 3D; p<0.01; Student's t test) and amygdala (Figure 3E; p<0.01; Student's t test) between MK-801-treated trained mice and vehicle-treated trained mice, as well as between trained mice and naïve mice (p<0.01; Student's t test). This concurrent effect on memory consolidation and post-training dephospho-eEF-2 strongly suggested a functional link between these two events, because the blockade of NMDA receptors was able to block both of them.

### Generation of hippocampus-specific eEF-2K transgenic (hip-eEF-2K-tg) mice

**Figure 4:**
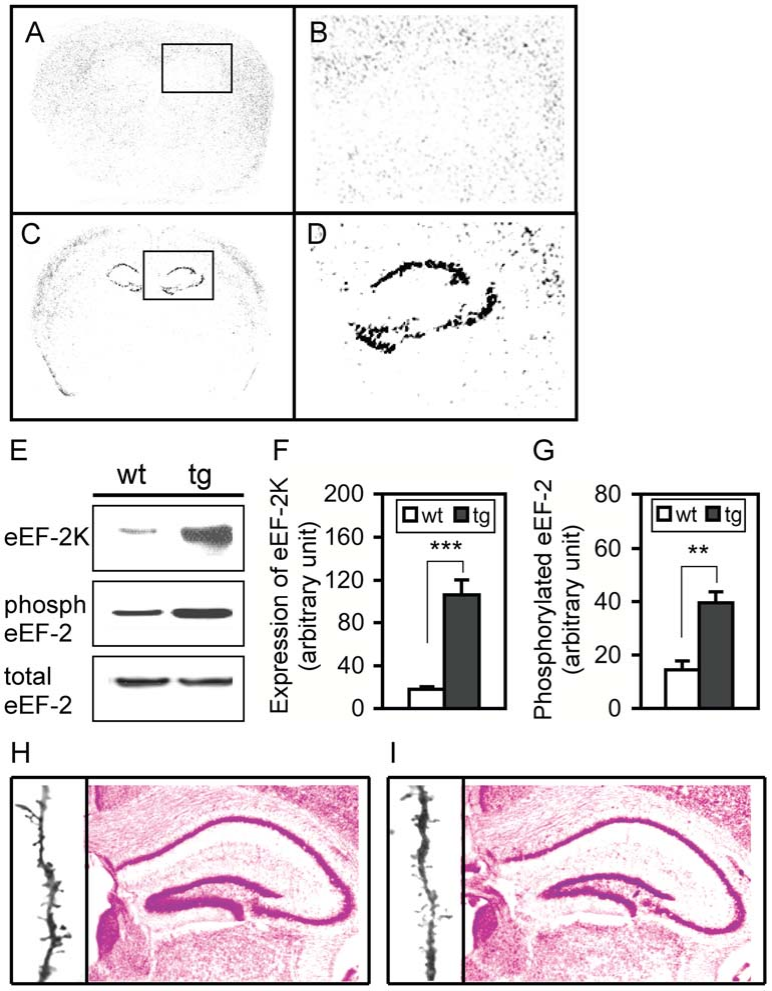
Generation of hip-eEF-2K-tg mice. A-D *in situ* hybridization showing the expression of the eEF-2K transgene in wild-type (A) and hip-eEF-2K-tg mice (C) or a higher magnification in wild-type (B) and hip-eEF-2K-tg mice (D). Exclusive expression of the transgene mRNA was found in the CA1, CA3, and dentate gyrus, but not the CA2 region. E. Representative Western blots showing the expression of the total eEF-2K (including the endogenous and transgenic eEF-2K; upper panel), phospho-eEF-2 (phosph) (middle panel), and the total eEF-2 (including phospho- and dephospho-eEF-2; low panel) in the hippocampi from wild-type (wt) and hip-eEF-2K-tg (tg) mice. F. Quantitative analysis of the expression of eEF-2K in wt (n = 6) and tg mice (n = 5). ***, p<0.001, Student's *t* test. G. Quantitative analysis of the expression of phospho-eEF-2 in wt (n = 5) and tg mice (n = 5). **, p<0.01, Student's *t* test. H and I. Representative microphotography of Nissl staining and Golgi staining in wild-type (h) and hip-eEF-2K-tg mice (i).

The functional link between the dephospho-eEF-2 and memory consolidation conferred an opportunity to further study whether a blockade of the dephospho-eEF-2 following training impaired memory consolidation via blocking protein synthesis. Based on the findings that (1) eEF-2K is the most important kinase that phosphorylates and inactivates eEF-2, (2) the only identified substrate for eEF-2K is eEF-2 [23], [26], and (3) training in the FCT in the mouse creates two types of conditionings that are respectively hippocampus-dependent and -independent, to overexpress eEF-2K in the hippocampus only would provide an ideal system to specifically analyze whether the post-training dephospho-eEF-2 plays a role in memory consolidation. Accordingly, a Cre/loxP recombination system and a transcriptional silencing strategy were used to generate hip-eEF-2K-tg mice. Two independent transgenic mouse strains, eEF-2K transgenic mice and Cre transgenic mice, were needed (Figure S1). The eEF-2K transgenic mice were featured by Cre recombination-dependent deletion of a transcriptional stop signal that located upstream of the eEF-2K transgene so that the transgene would only express in the cells or brain region where Cre expressed. Fortunately, as the Cre expression in our Cre transgenic mice was limited to neurons in most parts of the hippocampus, Cre/eEF-2K double transgenic mice exhibited hippocampus-specific eEF-2K overexpression, which was evidenced by *in situ* hybridization (Figure 4A to D). The highest level of the transgene mRNA was observed in the CA1/CA3 regions, a lower level in the dentate gyrus, and little expression in the CA2 region (Figure 4D). Real-time RT-PCR showed a 10-fold higher level of the total eEF-2K mRNAs (endogenous and transgenic eEF-2K) in the hippocampus of hip-eEF-2K-tg mice than in wild-type mice (data not shown), whereas Western blot analysis showed a 5.5-fold higher level of eEF-2K protein in the hippocampus of hip-eEF-2K-tg mice than in wild-type mice (Figure 4E, upper panel and F). Moreover, a significantly higher level (about 2.5-fold) of phospho-eEF-2 was observed in the hippocampus of hip-eEF-2K-tg mice, compared to that in wild-type mice (Figure 4E middle panel and Figure 4G). A tendency of decrease in the total eEF-2 expression was observed in hip-eEF-2K-tg mice (Figure 4E low panel) but was not statistically significant. In the cortex and amygdala, no observable difference in the expression of eEF-2K, phospho-eEF-2, or the total eEF-2 was found between wild-type and hip-eEF-2K-tg mice (data not shown). These results indicated that we successfully generated hip-eEF-2K-tg mice and the eEF-2K transgene was functional.

### Overall characterization of hip-eEF-2K-tg mice

To exclude a possibility that a random insertion of a transgene into the mouse genome might produce some unexpected effects, the general conditions of the transgenic mice were carefully examined. Hip-eEF-2K-tg mice showed normal viability without observable abnormality in growth, body size, and mating, eating, and general behaviors, compared to those in their wild-type littermates (data not shown). Nissl staining and Golgi-impregnated staining did not reveal an observable difference between wild-type (Figure 4H) and hip-eEF-2K-tg mice (Figure 4I). Open-field behaviors indexed by movement time, total distance traveled, and rearing numbers were indistinguishable between these mice (Figure S2A-C). These results indicated that overall hip-eEF-2K-tg mice were similar to their wild-type littermates.

### Hippocampus-specific overexpression of the eEF-2K transgene specifically inhibited protein synthesis in the hippocampus, but not any other brain regions

**Figure 5:**
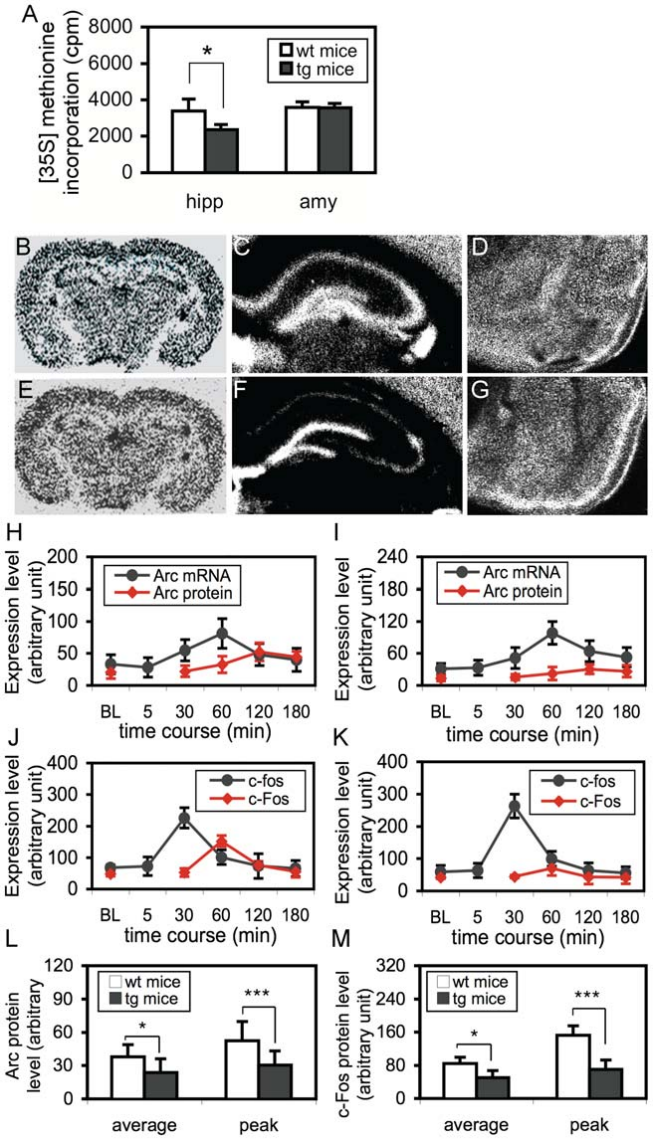
Protein synthesis inhibition in the hippocampus, but not amygdala, of hip-eEF-2K-tg mice. A. Quantitative analysis of [^35^S]-methionine incorporation into proteins in the hippocampi (hipp) and amygdalae (amy) from wild-type (wt; n = 6) and hip-eEF-2K-tg (tg) mice (n = 7). *, p<0.05, Student's *t* test. B and E. Representative autoradiography microphotographs showing protein synthesis inhibition in coronal brain sections from wild-type (B) and hip-eEF-2K-tg mice (E). C and F. A higher magnification of microphotographs showing protein synthesis inhibition in the hippocampus of hip-eEF-2K-tg mice (F), compared to wild-type mice (C). D and G. A higher magnification of microphotographs showing no observable protein synthesis inhibition in the amygdala of hip-eEF-2K-tg mice (G) compared to wild-type mice (D). H and I. Expression of Arc mRNA (black line) and Arc protein (red line) in the hippocampi from wild-type (H; n = 5 in each group) and hip-eEF-2K-tg mice (I; n = 5 in each group). BL: basal line from mice treated with vehicle. J and K. Expression of c-fos mRNA (black line) and c-Fos protein (red line) in the hippocampi from wild-type (J; n = 4 in each group) and hip-eEF-2K-tg mice (K; n = 4 in each group). BL: basal line from mice treated with vehicle. L and M. Quantitative analysis of the expression of Arc protein (L) and c-Fos protein (M) in hippocampi from mice after KA injection at the average level and peak level. *, p>0.05, Student's *t* test, ***, p<0.001, *post hoc* test, compared between wild-type (wt) and hip-eEF-2K-tg (tg) mice.

We then asked whether the increased phospho-eEF-2 affected protein synthesis. First, we used an *in vivo* [35S]-methionine labeling system [29] to examine the rate of [35S]-methionine incorporation into new proteins in the brain. As shown in Figure 5A, the [35S] incorporation in hippocampi from hip-eEF-2K-tg mice was slightly, but significantly, lower than in wild-type mice (p<0.05; Student's *t* test); whereas in either cortices (data not shown) or amygdalae (Figure 5A), no significant difference was found. In should be noted that as the assay was conducted with the lysates from the whole hippocampus that contained cells that expressed very little of the transgene (CA2 neurons) or that did not express the transgene at all (all glial cells), the results showed in Figure 5A did not represent the overall protein synthesis inhibition rate in all hippocampal neurons. This was also evidenced by autoradiography, which showed the lowest density of [35S]-labeling in the CA1/CA3 regions, a fairly low level in the dentate gyrus, and almost no change in the CA2 region of hip-eEF-2K-tg mice (Figure 5E and F), compared to those in wild-type mice (Figure 5B and C). Similarly, no observable changes could be detected in any other brain regions including the amygdala (Figure 5D and G) and cortex (Fig. 5B and E), confirming the hippocampus-specific effect. The pattern of the protein synthesis inhibition in different hippocampal sub-regions was almost the same as that in the transgene mRNA expression (Figure 4D). It should be noted that even in the CA1/CA3 regions where the transgene expression was at the highest level, the [35S]-labeling signal was still observable, indicating that protein synthesis was not completely diminished by the transgene.

We further employed a dynamic modeling system to evaluate the transgene effect. It has been well known that the expression of certain immediate-early genes such as Arc and c-fos can be quickly triggered by neuronal activities [30], [31]. To compare the difference between the mRNA transcription and protein translation in the same brain region of the same animal or between different animals provided a valuable means to analyze how the protein synthesis was specifically affected by the transgene. Accordingly, mice were either treated with vehicle (basal level, BL) or a single dose of kainic acid (KA; 20 mg/kg; i.p.), and then brains were collected with a time-course from 5 min to 3 hr. A robust induction of both Arc and c-fos mRNAs was observed in the hippocampus, with a peak at 60 min for Arc mRNA in either wild-type (Figure 5H) or hip-eEF-2K-tg mice (Figure 5I) and a peak at 30 min for c-fos mRNA in either wild-type (Figure 5J) or hip-eEF-2K-tg mice (Figure 5K). For a within-genotype analysis between vehicle- and KA-treated mice, we found a highly significant difference in Arc mRNA expression in either wild-type [F(5,25) = 6.24, p<0.001] or hip-eEF-2K-tg mice [F(5,25) = 10.34, p<0.001], and in c-fos mRNA expression in either wild-type [F(5,20) = 29.34, p<0.001] or hip-eEF-2K-tg mice [F(5,20) = 31.29, p<0.001]. A between-genotype analysis with a repeated ANOVA did not reveal a significant difference in the expression of either mRNA in these mice, indicating that the transgene did not affect the transcriptions of the mRNAs that were associated with neuronal activity. At the protein level, a delayed but similar expression pattern for both Arc and c-Fos was observed (red line; Figure 5H-K). In KA-treated wild-type mice, an one-way ANOVA confirmed the increase in both Arc expression [F(4,20) = 8.01, p<0.001] and c-Fos expression [F(4,15) = 37.88, p<0.001]. In KA-treated hip-eEF-2K-tg mice, the increase in the expression of either Arc [F(4,20) = 3.27, p<0.05] or c-Fos [F(4,15) = 4.65, p<0.05] was at a less significant level. Moreover, a repeated ANOVA revealed a highly significant difference in the expression of Arc [F(4,40) = 9.69, p<0.001] or c-Fos [F(4,30) = 39.79, p<0.001] between wild-type and hip-eEF-2K-tg mice, confirming the effect of the transgene on protein synthesis inhibition. The p values at the average level from four time-points of the time-course (p<0.05) and at the peak level (p<0.001) were different in either Arc (Figure 5L) or c-Fos expression (Figure 5M), indicating that the effect of the transgene was more robust during an “on-going” protein synthesis process.

### Expression of the eEF-2K transgene prevented post-training dephospho-eEF-2 in the hippocampus

**Figure 6:**
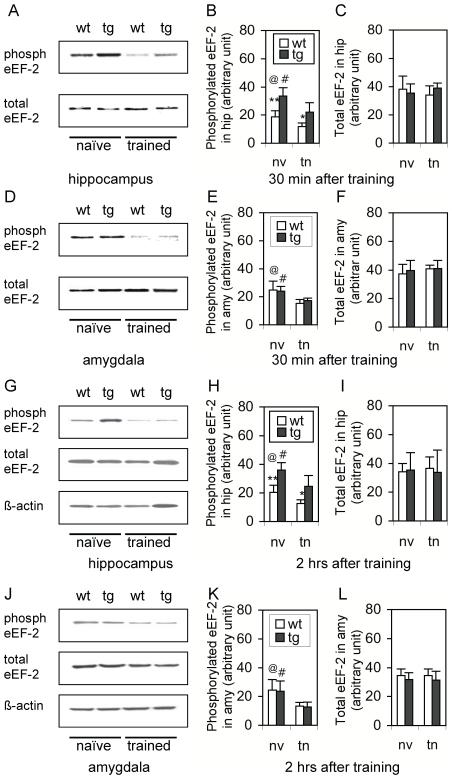
Overexpression of eEF-2K in the hippocampus prevented post-training dephospho-eEF-2. A-F. Expression of phospho-e-EF-2 in mice 30 min after the training. A. Representative Western blots showing the expression level of phospho-eEF-2 (phosph; upper panel) and total eEF-2 (low panel) in the hippocampi from wild-type (wt) and hip-eEF-2K-tg (tg) mice 30 min after training (trained) or without training (naïve). B. Quantitative analysis of the expression level of phospho-eEF-2 in hippocampi 30 min after the training. **, p<0.01, naïve wt mice vs. naïve tg mice; @, p<0.05, naïve wt mice vs. trained wt mice. #, p<0.05, naïve tg mice vs. trained tg mice; *, p<0.05, trained wt mice vs. trained tg mice. nv: naïve; tn: trained. C. Quantitative analysis of the expression level of the total eEF-2 in hippocampi 30 min after the training. D. Representative Western blots showing the expression level of phospho-eEF-2 (upper panel) and total eEF-2 (low panel) in the amygdalae of wt and tg mice 30 min after the training and without training. E. Quantitative analysis of the expression level of phospho-eEF-2 in amygdalae 30 min after the training. @, p<0.01, naïve wt mice vs. trained wt mice. #, p<0.05, naïve tg mice vs. trained tg mice. F. Quantitative analysis of the expression level of the total eEF-2 in amygdalae 30 min after the training. G-I. Expression of phospho-e-EF-2 in tg mice 2 hr after the training. G. Representative Western blots showing the expression level of phospho-eEF-2 (upper panel), total eEF-2 (middle panel), and β-actin (low panel) in hippocampi 2 hr after the training and without training. H. Quantitative analysis of the expression level of phospho-eEF-2 in hippocampi 2 hr after the training. **, p<0.01, naïve wt mice vs. naïve tg mice; @, p<0.05, naïve wt mice vs. trained wt mice. #, p<0.05, naïve tg mice vs. trained tg mice; *, p<0.05, trained wt mice vs. trained tg mice. I. Quantitative analysis of the expression level of the total eEF-2 in hippocampi of mice 2 hr after the training. J. Representative Western blots showing the expression level of phospho-eEF-2 (upper panel), total eEF-2 (middle panel), and β-actin (low panel) in amygdalae 2 hr after the training and without training. K. Quantitative analysis of the expression level of phospho-eEF-2 in amygdalae of mice 2 hr after the training. @, p<0.01, naïve wt mice vs. trained wt mice. #, p<0.05, naïve tg mice vs. trained tg mice. L. Quantitative analysis of the expression level of the total eEF-2 in amygdalae of mice 2 hr after the training. Sample size in each group was 4–5 mice, with at least 2 measures in each animal. Student's *t* test was used for all statistical analyses.

Before the training in the FCT, the phospho-eEF-2 level in the hippocampus (Figure 6A), but not the amygdala (Figure 6D), of hip-eEF-2K-tg mice was higher than that in wild-type mice. Thirty minutes after the training, the phospho-eEF-2 level significantly decreased (p<0.05; Student's *t* test) in both the hippocampus (Figure 6A and B) and amygdala (Figure 6D and E), without a significant change in the total eEF-2 level in either group (Figure 6C and F). Interestingly, a significant difference in the phospho-eEF-2 level was still observed in the hippocampus (Figure 6B and H; p<0.05; Student's *t* test), but not the amygdala (Figure 6E and K), between trained wild-type and trained hip-eEF-2K-tg mice, indicating that the effect was due to the transgene expression. Similar changes were observed in mice sacrificed at 2 hr after the training (Figure 6G-L). These results indicate that the transgene was able to largely, but not completely, prevent post-training dephospho-eEF-2 in the hippocampus, but not amygdala.

### Long-term hippocampus-dependent memory was specifically impaired in hip-eEF-2K-tg mice

**Figure 7:**
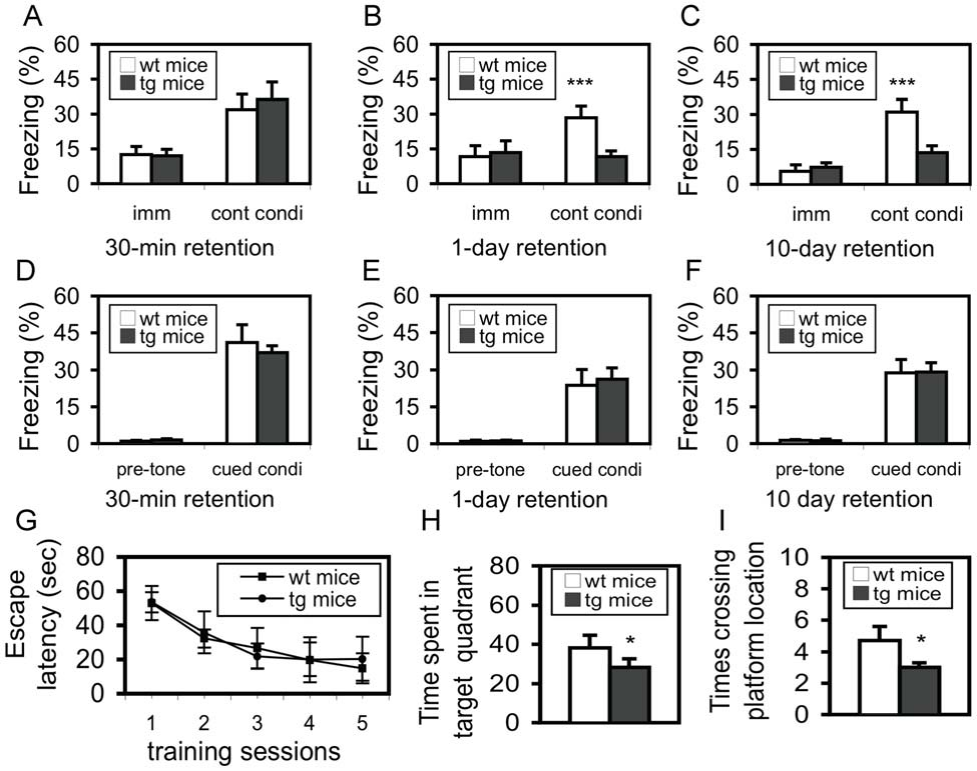
Long-term, but not short-term, hippocampus-dependent memory is impaired in hip-eEF-2K-tg mice. A. Short-term memory in contextual conditioning was measured at 30 min after the training. No significant difference was observed in either immediate (imm) freezing after shock or contextual conditioning (cont condi) between wild-type (wt, n = 10) and hip-eEF-2K-tg mice (tg, n = 13). B. Short-term memory in cued conditioning was measured at 30 min after the training. No significant difference was observed in either pre-tone freezing or cued conditioning (cued condi) between wt (n = 10) and tg mice (n = 13). C. Long-term memory in contextual conditioning was measured at 1 day after training. While no significant difference was observed in immediate freezing after shock, a highly significant difference was found in contextual conditioning between wt (n = 10) and tg mice (n = 12). ***, p<0.001, Student's *t* test. D. Long-term memory in cued conditioning was measured at 1 day after training. No significant difference was observed in either pre-tone freezing or cued conditioning. E. Long-term memory in contextual conditioning was measured at 10 days after the training. While no significant difference was observed in immediate freezing after shock, a highly significant difference was found in contextual conditioning between wt (n = 11) and tg mice (n = 12). ***, p<0.001, Student's *t* test. F. Long-term memory in cued conditioning was measured at 10 days after training. No significant difference was observed in either pre-tone freezing or cued conditioning. G. Learning curve in a water maze test; repeated ANOVA did not reveal a significant difference between wt (n = 11) and tg (n = 12) mice. H. Time spent in the target quadrant in a probe test 24 hr after the completion of the training sessions. *, p<0.05, Student's *t* test. i. Number of crossing over the platform location in the probe test 24 hr after the completion of the training session. *, p<0.05, Student's *t* test.

The results above validated an ideal system to test whether the post-training dephospho-eEF-2 was functionally associated with memory consolidation. In order to exclude a non-specific effect of the transgene on learning behavior, as well as to test whether the protein synthesis inhibition specifically affected long-term memory, both short-term and long-term memories were examined. At 30 min after the training, freezing response in contextual (Figure 7A) and cued conditioning (Figure 7D) was at the same level between wild-type and hip-eEF-2K-tg mice, indicating that the contextual and cued short-term memories were intact in hip-eEF-2K-tg mice. Long-term memory was examined at 1 day and 10 days after the training with two separate sets of mice. Compared to that in wild-type mice, a lower freezing rate was observed in eEF-2K-tg mice in contextual (Figure 7B and C; p<0.001; Student's *t* test), but not cued conditioning (Figure 7E and F), in both retention tests, indicating that long-term hippocampus-dependent memory, but not hippocampus-independent memory, was impaired. To exclude a possibility that a different nociceptive response might contribute to the difference above, the minimal amount of current required to produce stereotypical behaviors (flinching/running, jumping, and vocalizing) was measured after the retention tests, and the results did not show any significant difference between these mice (data not shown).

In order to determine whether the hippocampus-specific protein synthesis inhibition affected other types of hippocampus-dependent memory, spatial learning and memory were evaluated by using a Morris water-maze test [32], [33]. Mice were trained with a five-day training protocol. In both wild-type and hip-eEF-2K-tg mice, the escape latency dramatically decreased following the training (Figure 7G), and cross-sectional analyses with an one-way ANOVA revealed a highly significant difference in the latency in either wild-type [F(4,50) = 6.74, p<0.001] or hip-eEF-2K-tg mice [F(4,55) = 7.59, p<0.001]. Moreover, a repeated ANOVA did not reveal any significant difference between these two groups, indicating that all these mice could equally learn the task. In a probe test, however, a significant difference (p<0.05; Student's *t* test) in the amount of time spent in the target quadrant (Figure 7H) or in the number of crossing the area that represented the location of the platform previously placed during the training sessions (Figure 7I) was observed, indicating that the transgenic mice were impaired in spatial retention. Since the learning curve (training sessions) represents a compound effect of acquisition and retention, with a dominant influence from the acquisition, a normal learning curve in hip-eEF-2K-tg mice indicates an intact acquisition process. On the other hand, the probe test detects the “pure” retention of the spatial navigation, and a deficit in this test provides additional evidence that these transgenic mice are impaired in long-term hippocampus-dependent (spatial) memory.

### Late-phase LTP (L-LTP) was impaired in the hippocampus of hip-eEF-2K-tg mice

**Figure 8:**
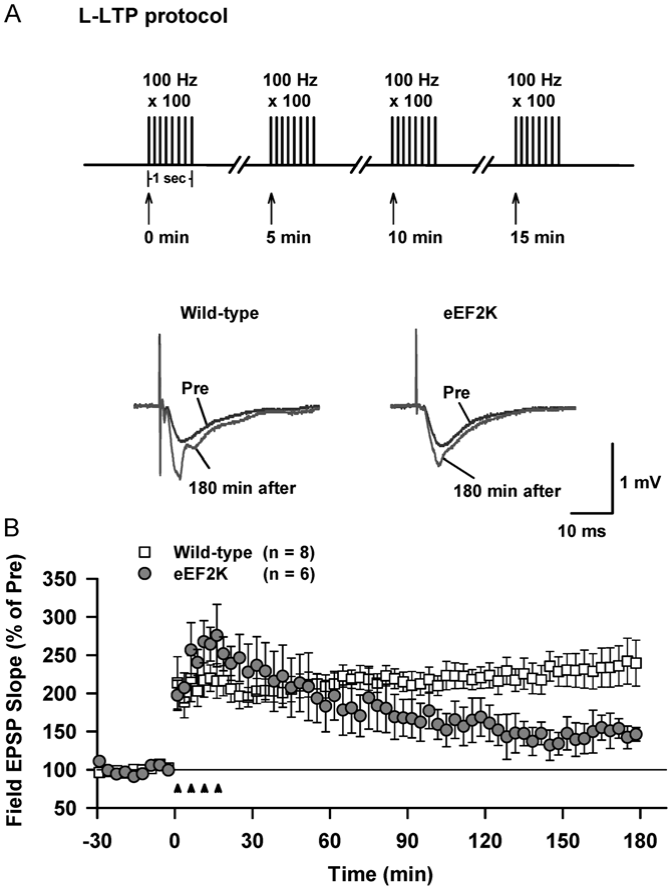
L-LTP, but not post-tetanic potentiation, is impaired in the hippocampus of hip-eEF-2K-tg mice. A. Four trains of high-frequency stimulation (100 Hz for 1 second) made the fEPSP still robust measured at 180 min after the stimulation in wild-type (wt) slices but not in transgenic (tg) slices. B. Quantitative analysis of the potentiation during this 180 min period indicated that there was no significant difference in LTP production (peak) and LTP maintenance before 90 min, whereas there was a highly significant difference exhibited from 90 min up to the whole observation period.

LTP, a cellular model for learning and memory, may also be divided into different phases. Evidence indicates that these different phases perfectly fit into memory stages [34]. Early-phase LTP, which is protein synthesis-independent, correlates to short-term memory, whereas L-LTP, which is protein synthesis-dependent, correlates to long-term memory [35], [36]. Therefore, it is important to examine whether the inhibition of protein synthesis affected L-LTP. As shown in Figure 8A, 4 trains of high-frequency stimulation (100 Hz for 1 second) were used to produce L-LTP in the hippocampal Schaffer collateral/commissural pathway. As shown in Figure 8B, it was apparently that the post-tetanic potentiation in hip-eEF-2K-tg slices was not significantly different from that in wild-type slices. This also indicated that the basal synaptic transmission was similar in all these mice. However, quantitative analyses revealed a highly significant difference between wild-type and hip-eEF-2K-tg slices from 90 min up to the whole observation period (180 min), whereas no significant difference was observed in LTP production (peak) or LTP maintenance before 90 min. These results indicated that L-LTP was specifically and significantly impaired in hip-eEF-2K-tg mice.

## Discussion

In this study, we first found that dephospho-eEF-2 in both the hippocampus and amygdala of mice was temporarily associated with post-training, whereas no any significant change in the expression of the mRNAs for translational machineries or their related molecules could be identified. The use of MK-801 then revealed that both post-training dephospho-eEF-2 and memory consolidation were neuronal activity-dependent, and that there was a functional link between these two events. At the last, by using a unique transgenic mouse model, we have documented that the post-training dephospho-eEF-2 is a molecular underpinning for protein synthesis pertinent to memory consolidation.

New protein synthesis is required for various forms of long-lasting synaptic plasticity. Although the exact mechanisms might be different in different forms of plasticity, protein synthesis itself is basically under the control of both the transcriptional and translational actions. At the transcriptional level, the expression of mRNAs may directly affect protein synthesis at a number of ways. For example, a changed expression level of the translational machineries or their related molecules such as a up-regulation of mTOR [37] or a down-regulation of eEF-2K [38] may facilitate the overall translational activity. However, our genome-wide screening study did not reveal any evidence to support that this is the case for memory consolidation. Another way is that the expression of certain mRNAs may directly lead to new protein synthesis. We indeed found that the expression of certain genes such as c-fos and BDNF was up regulated following the behavioral training, which is consistent with many other studies [39], [40]. Because the expression of c-fos and BDNF is critically involved in memory formation [41], [42], and because most of these up-regulated transcripts may be further processed for protein synthesis, the expression of these genes may certainly contribute to memory consolidation. However, evidence indicates that the expression of these non-translational machinery-related molecules is unable to fully explain how protein synthesis is regulated for memory consolidation [43], [44].

The finding that the training facilitates dephospho-eEF-2 sheds light on a new translational mechanism, which has been validated from several angles in this study. First, the dephospho-eEF-2 is coincided in the hippocampus and amygdala, both brain regions are importantly involved in consolidating fear memories [24]. Second, the post-training antagonism of NMDA receptors is able to concurrently block the dephosphorylation and memory consolidation, indicating a functional link between them. Third, as the NMDA receptor is the most important excitatory machinery in the brain, this concurrent effect indicates that both the post-training dephospho-eEF-2 and memory consolidation are neuronal activity-dependent, which is also supported by other reports [25], [45]–[47]. Fourth, blockade of the dephospho-eEF-2 by the eEF-2K transgene dramatically inhibits protein synthesis, and this effect is more robust during an ongoing protein synthesis process that is associated with neuronal activity (Figure 5). Given that both neuronal activity and protein synthesis are critically involved in memory consolidation [15], [24], [25], a stronger effect during an ongoing protein synthesis process than during the general conditions provides a basis to identify whether a dynamic process of protein synthesis is more importantly required for memory consolidation. Indeed, we found that while these transgenic mice looked undistinguishable from their wild-type littermates at the overall level (Figure S2), both L-LTP and long-term hippocampus-dependent memory were significantly impaired (Figure 7 and Figure 8). All these results indicate that the post-training dephospho-eEF-2 plays a critical role in triggering memory consolidation-associated protein synthesis.

It should be mentioned that previous studies have found that the phospho-eEF-2 occurs within 15 min following the activation of NMDA receptors, and this phosphorylation is accompanied by protein synthesis inhibition [45], [48]. In contrast, we found here that the antagonism of NMDA receptor led to phospho-eEF-2 at 2 hr after the treatment. Therefore, we suggest that there might be two distinct phases, an early phase of phospho-eEF-2 and a late phase of dephospho-eEF-2, following the activation of NMDA receptors. Indeed, a late phase of dephospho-eEF-2, together with an enhanced overall protein synthesis, was found in those previous studies too [45], [48]. This “two-phase theory” may explain why the phospho-eEF-2 and inhibition of protein synthesis were observed within 1 hr after LTP production in the hippocampus [49], while in our study, it is supposingly that the L-LTP (after 3 hr) should be featured by an increase in dephospho-eEF-2. Another issue is about the effect of phospho-eEF-2. Although the overall protein synthesis is inhibited after the phospho-eEF-2, the expression of several specific molecules including Ca^2+^-calmodulin-dependent kinase II (CaMK-II) [45], Arc, and c-Fos [49] is up-regulated, which is inconsistent to our results (Figure 5). Given that (1) the increase of CaMK-II is found at the synaptic level [48]; (2) the expression of Arc and c-Fos may facilitate transcriptions of other genes; and (3) the increase of the translation of Arc and c-Fos occurs at the early phase of the phospho-eEF-2, a “synaptic competition” theory[48] might explain for this discrepancy, that is the up-regulation of Arc and c-Fos in their studies may represent a competitive mechanism for a late phase enhancement of protein synthesis. In our transgenic mice, as the transgene is constitutively expressed and as the phospho-eEF-2 is persistently higher, this compensative mechanism no longer exists, and thus the translation of Arc and c-Fos is inhibited. Regarding why a protein synthesis inhibitor could block early-phase LTP [50], [51], while the deficit in our transgenic mice was only observed at the L-LTP, it might be due to a different neuronal activity between these different conditions. The use of a protein synthesis inhibitor is generally accompanied by a higher synaptic function [52], while in our transgenic mice, the synaptic function may be persistently lower due to the constitutive transgene expression and thus, we could not identify a significant effect on the early-phase LTP.

Another important insight from the current study is that our study has demonstrated the requirement of new protein synthesis for memory consolidation from different angles. As described above, the role of protein synthesis in memory consolidation was originally established based on pharmacological studies with protein synthesis inhibitors [14], [15], [53]. Because the inhibitors are able to break the “chain” of the protein synthesis reaction, protein synthesis cannot be completed, which, in turn, renders the process of consolidation impaired. Virtually, all of those studies were essentially looking at the “consequences after the chain is broken”, but could not look into “how the chain works under the normal condition”. Recently, studies focusing on translational mechanisms have revealed some new insights. For example, either pharmacologically [54], [55] or genetically [56] blocking mTOR in the animals impairs memory consolidation. However, those studies are essentially similar to the pharmacological studies described above since those studies could not identify an active process that triggers protein synthesis for memory consolidation. In contrast, dephospho-eEF-2 following the behavioral training represents an active molecular process in the brain, and blocking this process leads to impairments in both L-LTP and long-term memory formation.

Why eEF-2 is dephosphorylated following the behavioral training is still unclear. Based on the findings that dephospho-eEF-2 is neuronal activity-dependent [45]–[47], [57], together with our findings that antagonism of NMDA receptors prevents both dephospho-eEF-2 and memory consolidation, we have reasons to speculate that this dephosphorylation is learning-triggered neuronal activity-dependent. Previous studies did find that phosphorylation of mTOR and p70S6 kinase was increased following training, but they could not demonstrate whether the phosphorylation itself was essential, since they did not have an approach to specifically block the phosphorylation in the brain [58]. Based on the role of both eIF and eEF in protein synthesis, the dephospho-eEF-2 might not be the only mechanism. Recent evidence indeed indicated that phosphorylation/dephosphorylation of eIF was importantly involved in memory consolidation, while the results were inconsistent. Reduced phosphorylation of eIF2a by point mutation or by knocking out GCN2 (an eIF2a inhibitor) enhanced and impaired long-term memory, respectively [46], [59], [60], indicating that either the role of eIF is still unclear or that there may be a bi-directional role for phosphorylation of eIF in regulating protein synthesis [59]. In contrast, our studies have consistently shown the role of post-training dephospho-eEF-2 in memory consolidation from the behavioral, pharmacological, and genetic levels. Importantly, as eEF-2K-mediated phospho-eEF-2 is a major mechanism for the control of the rate of protein synthesis [23], post-training dephospho-eEF-2 may represent a fundamental mechanistic process that leads other translation mechanisms to promote protein synthesis for consolidation of newly learned information.

We need to point out that the effects of the transgene in the current study do not actually indicate the role of a single gene in memory consolidation, and do not essentially explore the role of a genetic basis for learning and memory in this study, as many other studies did [61], [62]. The high expression level of the transgene should not be a “physiological condition” in anyway. However, this high expression level could be used as a tool to prevent training-induced dephospho-eEF-2 in a specific brain region–the hippocampus. The results of this blockage suggested that there might be a mechanism that works at the network level to control the process of learning and memory, especially memory consolidation [63]. The answer to this question will be the next topic of our study.

## Materials and Methods

All the experiments for the use of mice were performed according to the protocols approved by the Animal Care and Use Committee in Louisiana State University Health Science Center at New Orleans, and conformed with National Institutes of Health guidelines.

### Fear-conditioning training

The procedures for training in FCT were the same as described previously [33]. Briefly, the conditioned stimulus (CS) was a tone at 90 dB and 2,800 Hz, and the unconditioned stimulus (US) was foot shock at 0.8 mA. During training, adult mice [2–3 months old; either B6/CBA F1 mice (from the Jackson Laboratory) or hip-eEF-2K-tg mice and their wild-type littermates] were individually put into the shock chamber and were allowed to freely explore the environment for 150 sec in the chamber. Afterward, the CS was delivered for 30 sec, and at the last 2 sec of the CS, the US was delivered. After the CS/US pairing, mice were allowed to staying in the chamber for another 30 sec and then were returned to their homecages.

### cDNA microarray

The procedures for cDNA microarrays were the same as described previously [64]. Briefly, three groups of adult (2–3 months old) B6/CBA F1 mice, together with a group of naïve control mice (the same strain; without shock, but with exposure to the shock chamber), were sacrificed at 30, 60, and 120 min after the training in the FCT, respectively. The total RNA was extracted from the hippocampi and amygdalae with Trizol (Invitrogen) and was purified with RNEasy columns (Qiagen). Reverse transcription (RT) was performed using the SuperScript® III First-Strand synthesis system (Invitrogen). All samples were hybridized in duplicate to Affymetrix 420 2.0 Array Chips, which was conducted by the Core Facility at the University of Chicago. The same cDNA microarray was repeated in three individual mice. The array expression values were generated using Affymetrix Microarray Suite 5.0 and dChip analyzer 1.3.

### Quantitative analysis of dephospho-eEF-2

Protein lysates were prepared from the hippocampi, amygdalae, and cortices of three groups of mice that were respectively sacrificed at 30 min, 2 hr, and 4 hr after the training in FCT. To exclude any non-specific effects, four control conditions were designed: naïve control (NC; mice were sacrificed without any treatment), shock control (SC; mice were sacrificed immediately after receiving the US), contextual control (CC; mice were sacrificed 30 min, 2 hr, or 4 hr after being exposed to the shock chamber for 5 min but without shock), and tone control (TC; mice were sacrificed 30 min, 2 hr, or 4 hr after being exposed to the CS). A total of 100 µg of protein lysates from each sample was separated by an SDS-PAGE (8%) and then was transferred onto Immobilon-P membranes (Millipore). The membranes were incubated with either anti-phospho-eEF-2 antibody (1∶2,000) or anti-total eEF-2 antibody (1∶2,000; all from Cell Signaling and Technology Laboratories), followed by HRP-conjugated secondary antibody (1∶4,000; Jackson ImmunoReseach). Blotting signal was visualized with the ECL detection system (Pierce). The same membranes were re-probed to anti-β-actin antibody (1∶10000, Santa Cruz Biotechnology Inc). Densitometry was performed using Image J Analysis software (version 1.39c). The phosphorylation ratio (%) was calculated as: (total eEF-2 - phospho-eEF-2)/total eEF-2 X 100. In order to minimize the artificial signal, the exposure time was the same for the membranes used to detect the total eEF-2 and phospho-eEF-2. The dephospho-eEF-2 ratio (%) after the training was calculated as: [phospho-eEF-2 of naive hippocampus (or amygdala) - phospho-eEF-2 of trained hippocampus (or amygdala)/phospho-eEF-2 of naive (or amygdala) X 100%]. The change in the total eEF-2 ratio was calculated as: [total eEF-2 of wild-type hippocampus (or amygdala) - total eEF-2 of transgenic hippocampus (or amygdala)/total eEF-2 of wild-type hippocampus (or amygdala) X 100%]. The expression level in each sample was normalized to the expression level of β-actin before the calculation. The quantitative data were the average of the levels from 5–6 mice, with at least 2 measures in each animal.

### NMDA receptor antagonism

An NMDA receptor antagonist, MK-801 (St. Louis, MO; 0.2 mg/kg), was administered (i.p.) immediately after the training in FCT. Both contextual and cued conditionings were examined at 2 hr or 24 hr after the MK-801 treatment with two separate sets of mice. The procedures for memory retention test are described below. Another three groups of mice (naïve/vehicle, training/MK-801, and training/vehicle) were used for immunoblotting experiments, in order to determine the phospho-eEF-2 level as described above.

### Generation of hip-eEF-2K-tg mice

An advanced transgenic approach was used in this study. Two independent transgenic mouse strains were needed. One was Cre transgenic mouse strain, in which the expression of the Cre recombinase was under the control of ∂-CaMK-II promoter. An 8.5 kb of ∂-CaMK-II promoter was used to drive a 2.6 kb of *Not I* transgene cassette that consisted of a 0.6 kb exon-intron splicing signal, a 0.4 kb element encoding a nuclear localization signal (pBS317), a 1.029 kb Cre cDNA, and a 0.6 kb poly-A signals (pNN265). All these components were subcloned into a pBS(−) vector. Due to some locus effects, the random insertion of ∂-CaMK-II promoter into the mouse genome could generate different expression patterns of the transgene. In our Cre transgenic mice, the expression of Cre was exclusively observed in most parts of the hippocampus. The other mouse strain was eEF-2K transgenic mice, in which the expression of the eEF-2K transgene was under the control of a chicken β-actin promoter so that the transgene would express in all types of cells. However, a transcriptional silencer (stop sequence) was put upstream of the eEF-2K cDNA to silence its transcription. Moreover, this stop sequence was flanked by two loxP elements. A recombination by the Cre recombinase led to the deletion of the stop sequence so that the expression of the eEF-2K transgene occurred in the brain regions where Cre expressed. The eEF-2K cDNA was cloned from the total RNA extracted from a brain of a B6/CBA F1 mouse with the primers of 5′-GCA ACA TGG CAG ACG AAG ACC TCA TC-3′ and 5′ GGG GCA GTT ATT CCT CCA TCT GGG CC-3′. The cDNA was confirmed by sequencing. The eEF-2K cDNA was flanked by an artificial intron and SV-40 poly-A signal, all from pNN265, in order to ensure a correct translation and to make the transgene distinguishable from the endogenous eEF-2K gene. Embryo donors and foster mothers were all from B6/CBA F1 mice (Jackson Laboratory), in order to (1) have a similar genomic background between transgenic mice and their wild-type littermates, (2) have a better genetic background for behavioral analysis, and (3) have a comparable genetic background to the non-transgenic studies as shown in Figure 2. After being linearized with appropriate restriction enzymes, the expression cassette was injected into the pro-nuclei of B6/CBA F1 zygotes to produce transgenic founders. The transgene copy number in the founders was determined by Southern blots (data not shown). In order to have a higher expression level of the eEF-2K transgene, a founder with a gene copy number of about 12 of the eEF-2K transgene was bred into Cre transgenic mice to produce double transgenic mice. The genotypes of mice were determined by PCR analyses of the genomic DNA from the tails, which respectively detected the Cre transgene and the eEF-2K transgene.

*in situ* hybridization. The procedures for *in situ* hybridization were the same as described previously [33]. Briefly, an oligo probe (5′- CAC CAC AGA AGT AAG GTT CCT TCA CAA AGA TCC TCT AGC-3′) that specifically recognized the eEF-2K transgene only was 35S-labeled. Coronal brain sections (20 µm) were made with a Cryostat (Leica, CM 1900), and the hybridization was the same as described in our previous publication [33]. After being washed, the brain sections were exposed to Kodak Hyperfilm™ MP film. The hybridization signal was visualized with an Olympus B X 51 TF microscope and analyzed with the Q-imaging system.

### Histology

The procedures for histological experiments were the same as described previously [65]. Briefly, mice were anesthetized with sodium pentobarbital (Sigma-Aldrich) and were perfused transcardially with 0.9% saline followed by 4% paraformaldehyde (PFA). Brains were removed and post-fixed overnight with 4% PFA in 30% sucrose. Coronal brain sections (40 µm) were made with the Cryostat and were then used for Nissl (cresyl violet) staining. For Golgi-impregnated staining, mice were anesthetized and perfused transcardially with 0.9% saline followed by 4% PFA. Brains were kept in Golgi-Cox solution with light-tighten for 6 days and then in 30% sucrose for another 2–3 days. Brains were mounted on sectioning stages with cyanocacrylic glue. A vibratome (Leica, VT1200) was used to make brain sections (200 µm), which were then mounted onto 2% gelatinized microscope slides. Once mounted, the blotted slides were kept in a humidity chamber until ready to be stained. For staining, the slides were placed in glass staining tray and processed as following: (1) Rinsed in distilled water for 1 min; (2) Placed in ammonium hydroxide for 30 min in the dark; (3) Rinsed in distilled water for 1 min; (4) Placed in Kodak Fix for film for 30 min in the dark; (5) Rinsed in distilled water for 1 min; and (6) Dehydration with 50% to 100% EtOH for a couple of times at each steps, and then were mounted with a cover-lip.

### Protein synthesis under the normal conditions

L-[35S]-methionine incorporation rate was examined, in order to evaluate protein synthesis. Both hip-eEF-2K-tg and wild-type littermate mice were treated (i.p.) with a single dose of L-[35S]-methionine (150 µCi/animal, specific activity 151 Ci/mmol, GE Healthcare), and were sacrificed 2 hr after the treatment by decapitation. For quantitative analysis, brain tissues including the hippocampus and cortex (control) were quickly dissected from brains and protein lysates were prepared for liquid scintillation analysis as described elsewhere [29]. The concentration of L-[35S]- methionine incorporation into proteins in these tissues was calculated based on tissue weight. To map the brain region-specific protein synthesis, serial coronal brain sections (20 µm) were made on the Cryostat and autoradiography of L-[35S]- methionine incorporation (exposed to Kodak Hyperfilm™ MP film, Amersham for 10 days) was conducted as described elsewhere [66]. The -[35S]- methionine incorporation signal was analyzed with an Olympus microscope (SZ-PT) and the Q-imaging system.

### Protein synthesis under the conditions of enhanced neuronal activity

To evoke neuronal activity in the hippocampus, both hip-eEF-2K-tg and wild-type mice were treated (i.p.) with a single dose of KA (Sigma; 20 mg/kg), a non-NMDA receptor agonist, and were divided into 5 sub-groups. Behavioral responses were observed in their homecages and these five groups of mice were then sacrificed at a time-course of 5, 30, 60 120, and 180 min after the KA-treatment. The total RNA and protein lysates were extracted from the whole hippocampi of mice. Real-time RT-PCR (Applied Biosystem, 7900th) was used to determine the mRNA expression level for Arc and c-fos. Two customized fluorescence-labeled probes that recognized Arc and c-fos, respectively, were used. After RT, each reaction contained 5 µl Taqman Universal PCR Mastermix in a total volume of 10 µl containing 1.25 µl RT production. PCR reactions were conducted under the condition of 95°C for 10 min followed by 40 cycles of 30 sec at 95°C and 1 min at 60°C. The expression levels for both Arc and c-fos were normalized with 18S rRNA level. Final quantitative analysis was based on two measures (duplicate samples) in each mouse with a total of at least 4 mice in each group. Western blots were used to determine the expression of Arc and c-Fos at the protein level as described above. The concentrations of the anti-Arc antibody and anti-c-Fos antibody were both at 1∶2000 and these antibodies were purchased from Cell Signaling Technology Inc. The amount of protein loading was normalized by β-actin immunoblotting. Quantitative analysis was based on two measures in each mouse and in each group at least 5 mice were examined. The comparison of the ratio between the mRNA expression and protein expression within the same animal as well as the comparison of the difference in this ratio between wild-type and hip-eEF-2K-tg mice would provide a valuable means to test the specific inhibition at the translational level.

### Short-term and long-term memory

Both FCT and water maze test were used to examine memory functions [33]. For the FCT, both wild-type and hip-eEF-2K-tg mice (2–3 months old, with both female and male mice mixed) were trained with a one-trial protocol. For short-term memory, a retention test was conducted 30 min after the training. Both contextual and cued conditionings were examined. For contextual conditioning, mice were individually put back into the chamber where they received CS/US pairing, and freezing responses were recorded for 5 min with a sampling method at an interval of 5 sec. For cued conditioning, mice were individually put into a novel chamber and 3 min later, the same tone that was used during the training session was delivered for 3 min. Freezing responses were recorded in both pre-tone and during-tone periods. Freezing was defined as no movement of any part of the body, except for respiration. For long-term memory, every test was the same as described above, except for that the intervals between the training and retention test were 1 day and 10 days, respectively, with the use of two separate sets of animals. For the Morris water-maze test, a water tank with 1 meter in diameter and a computerized video-tracking system were used. A training protocol of 5 training sessions was used. Each training session per day contained 4 trials, with each trial lasting for 60 sec. The order of the quadrants for each mouse being released into the water tank was randomly designed for each session. The interval between each two trials was about 1 hr. After each trial, mice were towel dried and were immediately returned to their homecages. Escape latency to the platform, swimming speed, and swimming path were automatically recorded and the data were analyzed by a Nodel navigation tracking system (EthoVision, Pro-Noldus). One probe test was conducted 24 hr after the completion of all training sessions. During the probe test, the platform was removed, and mice were individually allowed to swimming in the pool for 60 sec. Time spent in each quadrant and times crossing over the place that the platform was previously located during the training sessions were recorded to determine long-term spatial memory.

### Open-field behaviors in hip-eEF-2K-tg mice

Open-field behaviors were examined by using an automatic-recording open-field working station (MED Associates, Georgia, VT) as described in our previous publication [67]. Briefly, the open-field box was illuminated by a dim light (20 lux) and two sets of 16 pulse-modulated infrared photobeams were placed on opposite walls 2.5 cm apart to record X-Y ambulatory movements. Mouse behaviors in the box were computer-interfaced at a sampling rate of 100-ms resolution. Before testing, mice were transported into the behavioral room to adapt to the environment for at least 1 hr. Behavioral responses of mice in the box were recorded for 60 min. The total path length and rearing times were recorded automatically.

### Electrophysiology

The procedures for electrophysiological recording were described in our previous publication [68]. Briefly, transverse hippocampal slices (400 µm) were cut from brains of wild-type and hip-eEF-2K-tg mice (2–3 months old), kept submerged at 27°C–28°C, and superfused (1–2 ml/min) with oxygenated (95% O2, 5% CO2) artificial cerebrospinal fluid (ACSF). Bipolar tungsten stimulating electrodes were placed in the CA1&3 to stimulate the Schaffer collateral and commissural fibers, and extracellular field EPSPs (fEPSPs) were recorded with a glass microelectrode (2–3 MU, filled with 2 M NaCl) positioned in the hippocampus. Baseline stimulation frequency was 2 min-1, and the intensity of the 0.1 ms pulses was adjusted to evoke 35%–40% maximal fEPSPs. Tetanic LTP was induced by high-frequency stimulation in brief trains (100 Hz, 1 s) applied either as a single train or four trains separated by 5 min intervals. To reduce day-to-day variability, simultaneous recordings were obtained from two slices. Data were recorded from wild-type and hip-eEF-2K-tg slices. The experimenters were blind to the mouse genotype.

### Statistical Analyses

Dada was analyzed by Student's *t* test or one-way ANOVA followed by the Bonferroni *post hoc* test wherever it was appropriate. A p value that was less than 0.05 was considered significance.

## Supporting Information

Figure S1 Constructs for transgenic mice. A. Expression vector for the Cre transgenic mice, which consists of an 8.5 kb of α-CaMKII promoter and a 2.6 kb Not I fragment encoding Cre gene. B. Expression vector for eEF-2K transgenic mice, which consists of a chicken β-actin promoter (3.1 kb), a stop signal that is flanked by two loxP elements (1.5 kb) and an eEF-2K cDNA that is flanked by an artificial intron and SV-40 poly-A signal.
(1.44 MB TIF)

Figure S2 Open-field behaviors in hip-eEF-2K-tg mice. A. Total movement time. B. Total travel time. C. Rearing numbers. No significant difference was found in any of these indexes between wild-type (wt, n = 11) and hip-eEF-2K-tg (tg, n = 12) mice. Cent: center area; Peri: peripheral area.
(1.74 MB TIF)
