# Supplementary figures and images for: Post-Training Dephosphorylation of eEF-2 Promotes Protein Synthesis for Memory Consolidation
Source: PLoS One. 2009 Oct 13;4(10):e7424. doi: 10.1371/journal.pone.0007424 (PMC2757674; doi:10.1371/journal.pone.0007424)

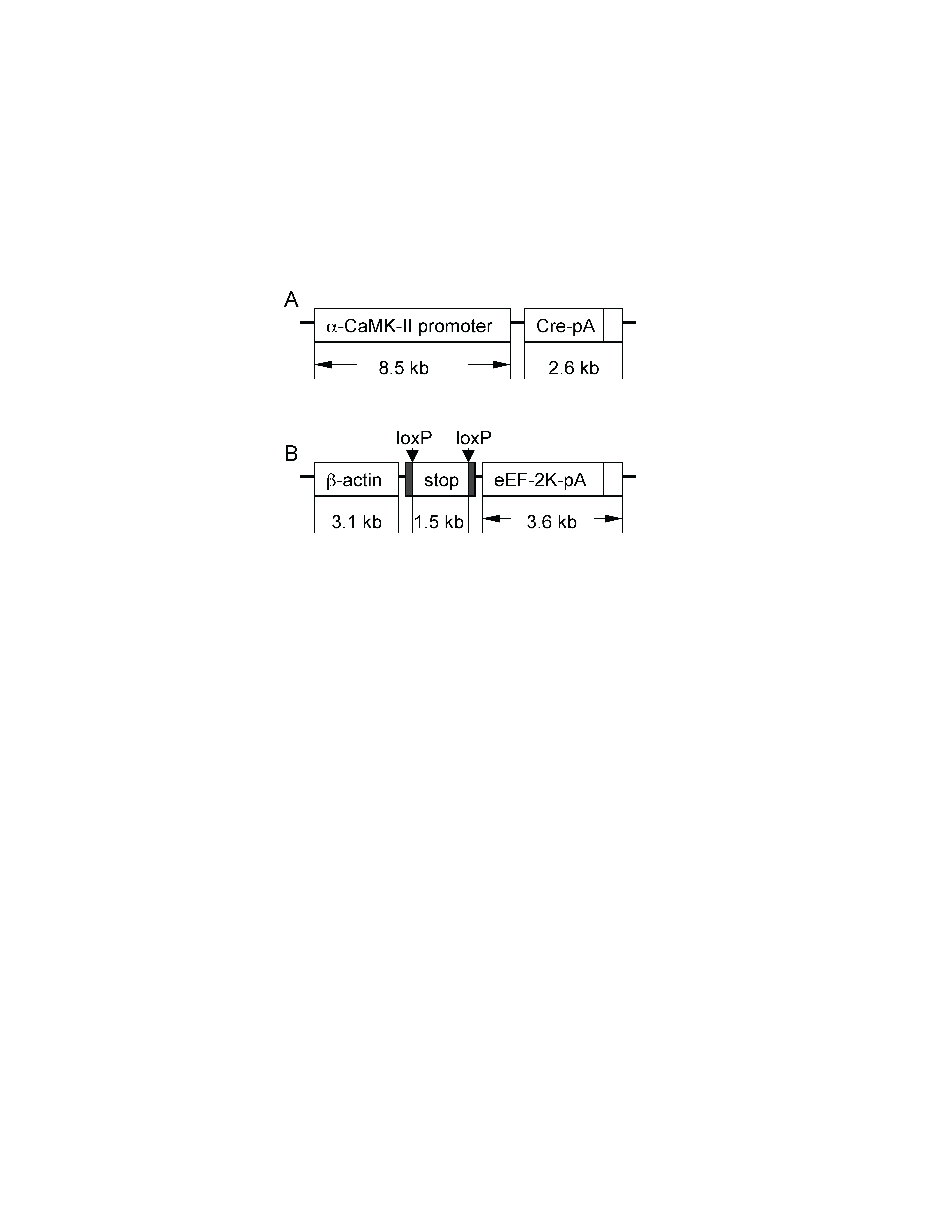

Supplement: Figure S1 — Constructs for transgenic mice. A. Expression vector for the Cre transgenic mice, which consists of an 8.5 kb of α-CaMKII promoter and a 2.6 kb Not I fragment encoding Cre gene. B. Expression vector for eEF-2K transgenic mice, which consists of a chicken β-actin promoter (3.1 kb), a stop signal that is flanked by two loxP elements (1.5 kb) and an eEF-2K cDNA that is flanked by an artificial intron and SV-40 poly-A signal. (1.44 MB TIF) [file pone.0007424.s001.tif]

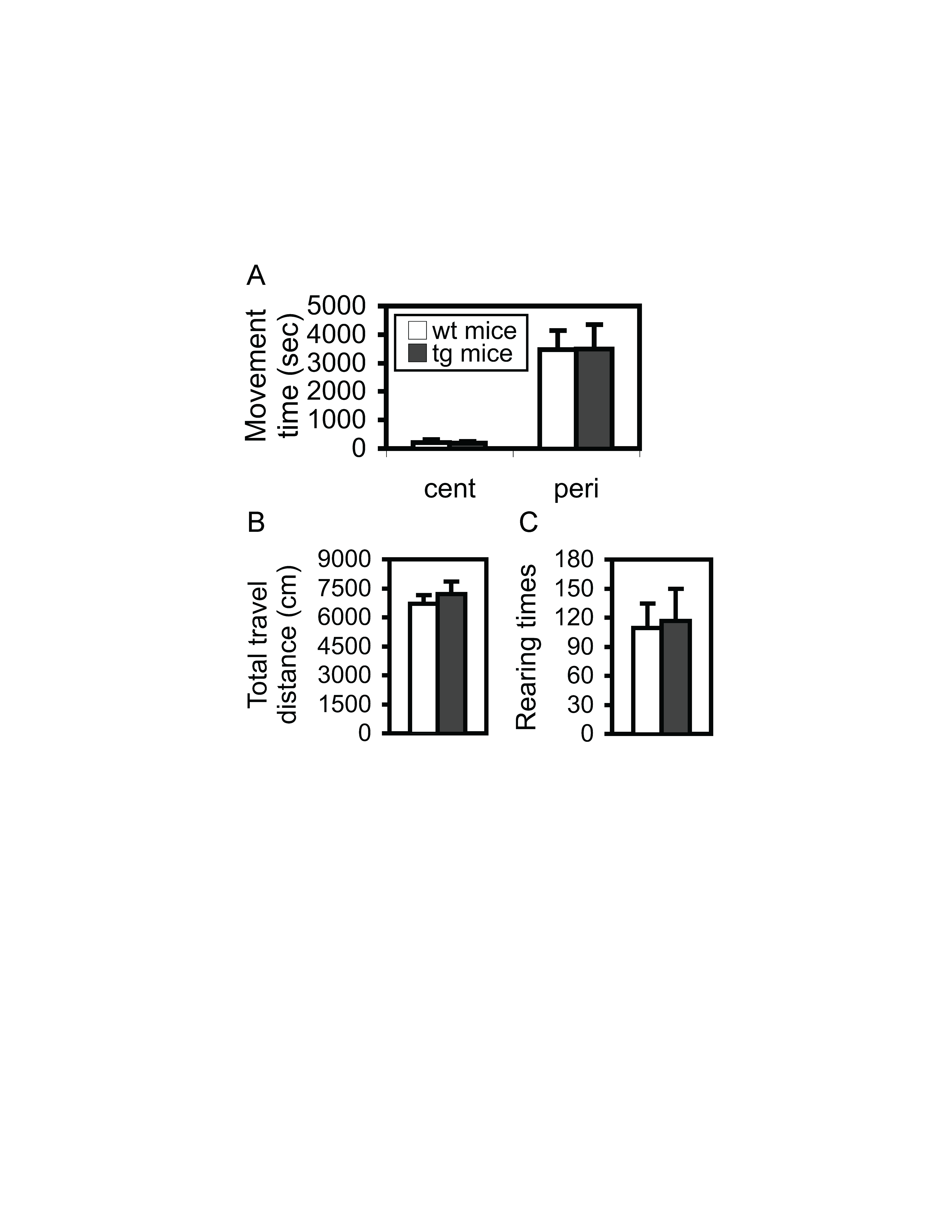

Supplement: Figure S2 — Open-field behaviors in hip-eEF-2K-tg mice. A. Total movement time. B. Total travel time. C. Rearing numbers. No significant difference was found in any of these indexes between wild-type (wt, n = 11) and hip-eEF-2K-tg (tg, n = 12) mice. Cent: center area; Peri: peripheral area. (1.74 MB TIF) [file pone.0007424.s002.tif]
